# Cryptococcal Neoformans and Varicella Zoster Meningitis in a Patient With Selective Innate Immunodeficiency: A Case Report

**DOI:** 10.7759/cureus.33490

**Published:** 2023-01-07

**Authors:** Sophiya Karki, Kenneth P Byrd

**Affiliations:** 1 Department of Medicine, University of Kansas Medical Center, Kansas, USA; 2 Division of Hematologic Malignancies and Cellular Therapeutics, Department of Internal Medicine, University of Kansas Medical Center, Kansas City, USA

**Keywords:** nk-cell deficiency, hypocomplementemia, immunodeficiency, immunosuppressed, hypereosinophilia, hypereosinophilic syndrome, varicella meningitis, cryptococcus meningitis

## Abstract

Cryptococcal neoformans (C. neoformans) and varicella-zoster (VZV) meningitis are opportunistic infections that are primarily seen in immunocompromised patients, including those with HIV, cancer, or receiving transplants. Despite treatment, infection in immunocompromised patients can be lethal, including those with T-cell dysfunction or deficiency. Whether innate immunodeficiencies also predispose to these infections remains less clear. Here, we report a case of disseminated C. neoformans and VZV meningitis in a young male with idiopathic hypereosinophilic syndrome and hypocomplementemia and no history of HIV infection, malignancy, or transplant. The patient presented with a pulsating headache, myalgia, joint pain, insomnia, night sweats, and subjective fever, along with clusters of vesicular lesions on his neck and back. A lumbar puncture and an MRI of the brain confirmed C. neoformans and VZV meningitis. Vesicular skin lesions proved to be VZV, and blood culture confirmed fungemia, suggesting disseminated disease. We investigated his medical history further to determine the underlying cause of his prior hypereosinophilia and current meningitis. The patient had idiopathic hypereosinophilia with high IgE levels, low complement levels, high rheumatoid factor levels, and an intermittent rash dating back two years, which had been treated intermittently with prednisone and hydroxyurea, with the most recent admission three weeks prior to this admission. Prior to admission, the patient had a peak absolute eosinophil count of 18.6 x10^3^/uL. The patient was discharged on a daily dose of 60 mg of prednisone without hydroxyurea. In further evaluating his immune status, we found he was HIV-negative, with a normal CD4 count and high IgE. We also tested lymphocyte subsets and proliferation, which showed a low CD16/56 level, suggesting possibly reduced natural killer (NK) cell quantity. The patient responded well to acyclovir, amphotericin, and flucytosine therapy. After follow-up cerebrospinal fluid (CSF) and blood cultures were negative, the patient was discharged with fluconazole as maintenance therapy.

## Introduction

Cryptococcus neoformans (C. neoformans) is an encapsulated yeast that is ubiquitous in the environment. However, in immunocompromised patients such as those with advanced Human Immunodeficiency Virus-Acquired Immunodeficiency Syndrome (HIV-AIDS), various T cell defects, inborn errors of immunity, malignancy, and transplant recipients, it can cause deadly meningitis [1]. Meningitis in HIV patients typically presents with a cluster of differentiation 4 (CD4) count of fewer than 50 cells/uL [2,3]. Despite treatment, C. neoformans meningoencephalitis in immunocompromised patients can be lethal, with a 10-week mortality of 15%-26% in the US [4]. The primary immune response to the fungus is CD4 and CD8 T cell-mediated [5,6]. Although eosinophilia is uncommon in cryptococcal infection, eosinophils appear to have a protective role in the disseminated form of cryptococcosis, with some basic research showing an allergic reaction to the fungus [7]. The impact of innate immune responses on fungal clearance is less clear. In vitro studies have shown that complement signaling (C5a-C5aR) in neutrophils is critical for killing C. neoformans [7]. In one study, complement components were found to be significantly elevated in the cerebrospinal fluid (CSF) of HIV-negative patients infected with C. neoformans [8]. Similarly, natural killer (NK) cells from the YT cell line were found to directly recognize b-1,3-glucan, a component of C. neoformans' cell wall, and mediate cytotoxic killing in vitro [9]. Varicella-zoster virus (VZV), like C. neoformans, causes high-mortality rates of meningoencephalitis in immunocompromised patients [10]. The severity of VZV infection has been associated with T and NK cell disorders, where the disseminated disease is associated with NK cell deficiency [11]. VZV can occur in adults with HIV at any CD4 T cell count, but the risk of infection is higher at a CD4 count< 200 cells/uL. Interestingly, eosinopenia has also been associated with a more serious condition, with higher morbidity and slow recovery [12]. Here, we report a case of C. neoformans and VZV meningitis that likely resulted from low complement and natural killer cell levels and possible eosinopenia due to prednisone use.

## Case presentation

A 27-year-old Hispanic man presented to the emergency department with a 10-day history of pulsating headaches, myalgia, joint pain, insomnia, night sweats, and subjective fever. The patient has a history of idiopathic hypereosinophilia dating back two years. In 2020, he had an anaphylactic-type episode that included rash, fever, abdominal pain, and hematochezia. That is when he was first found to have eosinophilia with a presenting absolute eosinophil count of 0.6x10^3/uL (ref: 0-0.45 10^3/uL). His rash was originally maculopapular but evolved into an annular erythematous rash. He was found to have elevated IgE and low complement levels. He was successfully treated with corticosteroids, epinephrine, and diphenhydramine. Over the next two years, he was monitored only intermittently due to non-compliance. However, he continued to have hypereosinophilia, with a count of 9.5x10^3/uL in February 2021 and a peak of 18.6 x10^3 /uL in April 2022. He was intermittently treated with steroids, and prior to this most recent admission, he had been on hydroxyurea. However, he stopped the hydroxyurea a couple of weeks prior to admission due to a worsening skin rash. Over the course of his hypereosinophilia, he followed up with hematology and rheumatology. A bone marrow biopsy in 2020 and again in 2022 showed no evidence of malignancy with normal cytogenetics and a hematologic Next-Generation Sequencing panel without pathogenic mutations. Fluorescent in situ hybridization (FISH) testing for platelet-derived growth factor receptors alpha and beta and fibroblast growth factor receptor 1 was normal. The tryptase level was normal. Infectious evaluations, including Strongyloides, stool ova, and parasites, were all normal. The PET/CT scan was unremarkable for malignancy. Per rheumatology evaluation, he had no clearly defined rheumatologic condition, although hypocomplementemic urticarial vasculitis syndrome was a working diagnosis. The plan was to further assess that with a skin biopsy upon recurrence, but that has not been possible to date. The patient did not have any family history of immunodeficiency.

On admission, the patient was afebrile but had a severe headache, leukocytosis (white blood count: 14x10^3/uL: ref: 4.5-11x10^3/uL), and generalized myalgia and joint pain. On physical exam, the patient seemed lethargic, diaphoretic, and in acute distress. Based on his symptoms, suspicion was high for meningitis. The patient was started on cefepime 2 mg IV, metronidazole 500 mg IV, and vancomycin 1000 mg IV on an empirical basis.

The brain MRI showed mild leptomeningeal enhancement without any focal lesions, further suggesting meningitis. A chest CT without contrast showed mild mediastinal and upper abdominal lymphadenopathy. Urinalysis was negative. These findings pointed to meningitis as the primary diagnosis.

**Figure 1:**
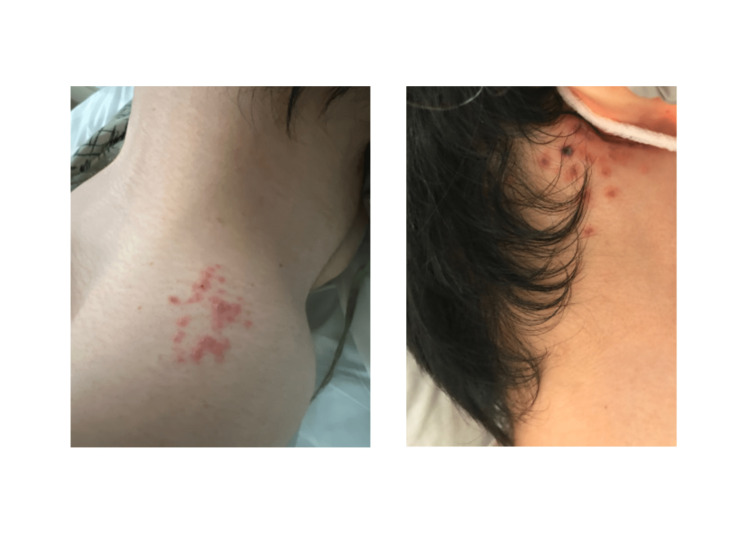
A vesicular rash on the left shoulder (left) and posterior aspect of the right ear (right). A skin swab tested positive for VZV.

On day two of admission, the patient developed an abrupt onset right-sided post-auricular vesicular rash concerning VZV (Figure 1). Another cluster was noted on the left shoulder a day later (Figure 1). Treatment was adjusted to target VZV by adding acyclovir 10 mg/kg and dexamethasone 10 mg IV daily to the treatment regimen. For the headache, the patient was given acetaminophen and oxycodone.

On day three, a lumbar puncture was done to confirm meningitis, and the infectious disease team was consulted for further recommendations. Cerebrospinal fluid (CSF) testing came back positive for C. neoformans antigen and VZV DNA, with no signs of lymphoma, bacterial infection (Table 1, negative gram stain), herpes simplex virus (HSV), or enterovirus infection. CSF studies were consistent with fungal and viral meningitis with an elevated white blood cell count, normal neutrophil count, and normal glucose, without xanthochromia. The patient also had right-sided cervical lymphadenopathy, with biopsy results that were unremarkable for leukemia and lymphoma but exceptional for cryptococcal species. PET scans showed hypermetabolic right cervical and mediastinal lymph nodes, consistent with biopsy results. Cryptococcal Ag also tested positive in blood culture, and a skin swab of the vesicular rash confirmed VZV infection, suggesting an overall disseminated disease (Table 1). Treatment was immediately adjusted to amphotericin B liposomal 3 mg/kg every 24 hours and flucytosine 25 g/kg every six hours, with the continuation of acyclovir. Prior to admission, the patient had been on prednisone 50 mg daily for his hypereosinophilic syndrome, which was slowly tapered off during his hospital stay. The patient’s absolute eosinophil count (AEC) on day three was 0.07 x10^3 /uL.

**Table 1:** Assays ordered during the hospital course HES: hyper-eosinophilic syndrome; AEC: absolute eosinophilic count; VZV: varicella-zoster; ref: reference

Cerebrospinal fluid (CSF)	Day 3	Day 5-8	Day 17
Herpes simplex virus 1 and 2 PCR	Neg		
VZV PCR	Positive		Not detected
Cryptococcal Ag	Positive (1:20)		Positive (1:5)
Enterovirus PCR	Neg		
Cell count and CSF test			
Red blood cells (cells/uL)	70		19
White blood cells (ref < 5 cells/uL)	30		17
Neutrophils (%)	51		
Lymphocytes (%)	42		95
Monocytes (%)	7		5
Clarity	Clear		Clear
Xanthochromia	None		None
Malignant cells	Negative		
Blood			
HIV 1 and 2 Ab	Neg		
Cryptococcal Ag	Positive (1:5)		
Microbiology			
Gram stain-CSF	No organisms		No organisms
CSF-culture	C. neoformans	C. neoformans	No growth
Blood-culture	C. neoformans	No growth	
VZV skin swab	Positive		
HES*			
AEC in blood** (0.05-0.5x10^3^/uL)	0.07	0.22	0.3
IgE in blood (<101 IU/mL)	547		
Hypo-complementamia			
C3 in blood (ref 88-200 mg/dL)	39		
C4 in blood (ref 10-49 mg/dL)	<8		
CH50 in blood (ref 41-95 U/mL)	<13		
Response to vaccine			
Tetanus IgG	Positive		
Pneumococcal IgG	Neg		
Diphtheria IgG	Positive		

Cryptococcal and VZV meningitis in a young patient presenting with flu-like symptoms prompted us to evaluate the patient for acute HIV infection and assess his CD4 count. HIV testing was negative, and the CD4 count was within the normal range (Table 2).

The allergy and immunology team was consulted to investigate any innate or adaptive immunodeficiency that would explain the patient’s presentation. A lymphocyte panel revealed viable lymphocytes with normal CD4 and CD8 T cell quantities and intact proliferative capacity in the presence of mitogens (Table 2). While the T cell proliferative response to Candida was normal, the response to the tetanus toxoid was diminished. Total immunoglobulin levels and IgG subtypes were unremarkable. (Table 2). Immunoglobulin E (IgE) levels remained consistently elevated; however, absolute eosinophil counts responded well to prednisone. IgG titers to tetanus toxoid and diphtheria vaccines were positive (Table 1), suggesting an intact humoral response. Interestingly, the NK cell count was only 37/uL, which was less than half of normal (Table 2). Complementary components C3, C4, and CH50 were very low during this admission (Table 1).

**Table 2:** Immunologic workup PWM: pokeweed mitogen; PHA: phytohemagglutinin

Lymphocyte panel	Values	Ref
% CD3	43.1	49-84 %
CD3 (count/mL)	609	600-2990
% CD8	9.8	10-40 %
CD8 (count/mL)	139	120-1320
% CD4	36	28-63%
CD4 (count/mL)	884	440-2160
CD19%	46	6-27%
CD19 (count/mL)	742	100-700
CD16/56% (NK cells)	2.6	4-25%
CD16/56 (count/mL)	37	90-640
Immunoglobulin		
Total IgG (mg/dL)	877	767-1590
IgG1 (mg/dL)	439	341-894
IgG2 (mg/dL)	144	171-632
IgG3 (mg/dL)	131	18.4-106
IgG4 (mg/dL)	155	2.4-121
CCP-IgG (U/mL)	<0.5	<0.5
IgM (mg/dL)	123	38-328
IgA (mg/dL)	99	70-390
Max proliferation with mitogens		
CD3 PWM	34.30%	>=3.5
CD19 PWM	18.9	>=3.9
CD45 PWM	25.60%	>=4.5
CD3 PHA	82.9	>=58.5
CD45 PHA	78.7	>=49.9
Max proliferation in response to Candida % CD3	8.8	>=3
Max proliferation in response to Candida % CD45	6.4	>=5.7
Max proliferation in response to tetanus toxoid %CD3	0.1	>=3.3
Max proliferation in response to tetanus toxoid %CD45	0.1	>=5.2
Viability of lymphocytes	75.4	>=75%

The patient’s persistent joint complaints and known history of hypocomplementemia prompted us to also consult rheumatology for any underlying rheumatological condition. The autoimmunity antibody panel, including antinuclear antigen (ANA), dsDNA, anti-SSA/SSB, MPO/PR3, and anti-CCP, were all negative except for rheumatoid factor (RF), which had been persistently elevated (Table 3).

**Table 3:** Rheumatologic workup RA: rheumatoid arthritis; RF: rheumatoid factor; anti-CCP: anti-cyclic citrullinated peptide; anti-SSA/SSB: anti-Sjogren's syndrome; anti-dsDNA: anti-double-stranded DNA; anti-ANA: anti-nuclear antibody; MPO/PR3: myloperoxidase/proteinase 3; SLE: systemic lupus erythmatosus

Condition	Antibodies	Antibody/titer
RA, SLE, Sjogren's	RF	728
RA	anti-CCP	Neg
Sjogren's, SLE	anti-SSA/SSB	Neg
SLE	anti-dsDNA	Neg
Many	anti-ANA	Neg
Systemic vasculitis	MPO/PR3	Neg

By day five, blood cultures were negative for C. neoformans, whereas CSF cultures still tested positive (Table 1).

By day 17, CSF culture was negative for C. neoformans, with a low detectable titer of antigen. VZV was also no longer detected in CSF (Table 1). The patient's leukocytosis had subsided, and his headache had also subsided. The patient was discharged on fluconazole (600 mg/day) maintenance therapy for 12 weeks. A recommendation for revaccination was made due to a poor T-cell response to tetanus toxoid. The patient was reported to be doing well at the six-week follow-up, and it was recommended that he switch to 200 mg/day in four weeks, to be continued for six to 12 months. Further immunologic genetic testing was planned as an outpatient procedure.

## Discussion

This case report highlights that susceptibility to C. neoformans and VZV meningitis is complex and that innate immunity may have a more important role in resisting these infections than previously appreciated. This patient had no history of HIV, malignancy, or transplant, and testing showed normal T and B cell numbers and functions, normal immunoglobulin levels, and a normal response to tetanus and diphtheria vaccines, although the proliferative response to the pneumococcal vaccine was selectively diminished. The most relevant medical history that could be tied to his presentation was his recent diagnosis of idiopathic hypereosinophilic syndrome and hypocomplementemia. Some studies have highlighted the important role of eosinophils in C. neoformans clearance [13]. Prior to admission, the patient was on 50 mg of prednisone daily for his hypereosinophilic syndrome and showed an eosinophil count of 0.07 x 10^3 cells/mL, which is on the lower end of normal. It is possible that a suboptimal eosinophil response renders patients susceptible to C. neoformans. On the other hand, it has been described previously that hypereosinophilia can be seen in the setting of primary immunodeficiency [14]. Notably, IgE levels were persistently elevated during this admission. It's unclear whether this rise is due to previous hypereosinophilia or a reaction to the fungi. Without antigen-specific assays, it is hard to delineate the role of IgE in this scenario.

In terms of innate components, complement signaling appears to be critical for an effective neutrophil response to fungal infection [7]. Low C3, C4, and CH50 levels noted in this patient in the absence of autoantibodies (Table 3) suggest that the patient’s presentation is less likely to be rheumatological and more associated with idiopathic hypocomplementemia. The significance of a positive rheumatoid factor in this case is unclear. No evidence of malignancy in the CSF, bone marrow, or on PET and CT scans suggested that the patient had no underlying malignancy that would explain his susceptibility to these infections.

Not much is known about innate immune deficiencies that could render one susceptible to VZV. However, a recent study associated NK disorders with severe disseminated VZV infection, which suggests that relative NK deficiency is a potential cause of VZV susceptibility in this patient [12]. Furthermore, the International Union of Immunological Societies (IUIS) committee on Inborn Errors of Immunity (IUI), which publishes phenotypic classifications of IUI with associated genetic classification, categorized STAT1 gain-of-function mutations in intrinsic and innate immunity with a predisposition to viral and fungal infections and STAT5b mutation with hypereosinophilia [15]. Therefore, an innate immunodeficiency gene panel, as suggested by IUIS, would enlighten this case.

Overall, the dysregulation of multiple immune components in this patient makes it challenging to pinpoint which component is more important for these infections. Furthermore, simultaneous infection with C. neoformans and VZV makes it challenging to assess whether susceptibility is specific to one pathogen or generalized. In addition, in vitro studies are hard to translate to these rare clinical scenarios. From a clinical standpoint, it would be useful to know whether the prognosis of C. neoformans and VZV meningitis is better in patients with innate immunodeficiency than in those with defective T-cell immunity. We hope that this case report inspires the scientific and medical communities to take a closer look at innate immunodeficiencies as they relate to susceptibility to potentially lethal infections.

## Conclusions

To the best of our knowledge, this is the first report of disseminated C. neoformans with concomitant VZV meningitis. Reviewing various aspects of this case, it is clear that this patient was immunocompromised, likely partially acquired from steroid use. Moreover, low NK cells and low complement levels suggested that this patient likely also had innate immune dysfunction in the setting of hypereosinophilic syndrome. The patient was promptly treated with intensive antimicrobials, which led to a complete recovery.
